# Secular Growth Trends in Early Childhood—Evidence from Two Low-Income Birth Cohorts Recruited over a Decade in Vellore, India

**DOI:** 10.4269/ajtmh.21-0886

**Published:** 2022-07-13

**Authors:** Beena Koshy, Arun S. Karthikeyan, Venkata Raghava Mohan, Anuradha Bose, Sushil John, Gagandeep Kang

**Affiliations:** ^1^ Developmental Paediatrics Unit, Christian Medical College, Vellore, India;; ^2^ Wellcome Research Unit, Christian Medical College, Vellore, India;; ^3^ Community Health, Christian Medical College, Vellore, India;; ^4^ Low Cost Effective Care Unit, Christian Medical College, Vellore, India

## Abstract

Stunting and extreme poverty are considered significant risk factors impacting child development in low-and-middle-income countries. We used two birth cohorts recruited 8–9 years apart in urban low-income (slum) settings in Vellore, south India and analyzed secular growth trends and their predictors. In the rotavirus cohort recruited between 2002 and 2003, 373 children completed the 3-year follow-up. “The Etiology, Risk Factors and Interactions of Enteric Infections and Malnutrition and the Consequences for Child Health and Development” (MAL-ED) cohort recruited between 2010 and 2012 had 215 children completing follow-up. The MAL-ED cohort had better socio-economic status (SES) markers and mothers were better educated compared with the previous cohort. Children in the MAL-ED cohort had less stunting at 1, 2, and 3 years of age. The linear mixed effects model evaluating linear growth during the first 3 years of age showed that low birth weight and being a female child were associated with stunting in both cohorts. There was no association between SES and stunting in the rotavirus cohort, whereas SES was associated with linear growth in the MAL-ED cohort. Future studies could incorporate nutritional and nonnutritional interventions in vulnerable populations to evaluate their effect on birth weight as well as early childhood stunting.

## INTRODUCTION

Stunting and poverty are risks impacting child development in low-and-middle-income countries (LMIC).^1^ Stunting causes not only increased mortality and morbidity in children, but also elevated risk of adult metabolic disease and poorer cognitive ability; affecting individual and consecutively national economic potential.^2^ Stunting can perpetuate the intergenerational cycle of reduced human potential, as stunted mothers usually deliver growth-restricted babies. The term “stunting syndrome” has been suggested to encompass this lifetime and intergenerational impact of stunting on overall individual potential.^2^

It is estimated that stunting affected around 150 million under-five children globally in 2018, and is more prevalent in south Asian countries,^3^ with a declining prevalence over the last two decades.^1^ Risk assessment analysis of childhood stunting, utilizing pooled data from 137 developing countries, showed fetal growth restriction and unimproved sanitation as leading risks worldwide.^4^ Other associated risk factors include parental height,^5^^–^^7^ socio-economic factors,^6^^–^^8^ pregnancy duration,^9^ diarrhea incidence,^9^^–^^11^ high entero-pathogen exposure in early childhood,^7^^,^^12^ poor child dietary diversity,^9^ and low energy and protein dietary intake.^7^^,^^12^

Recent meta-analysis of long-term implications of early childhood stunting, utilizing 425 LMIC birth cohorts, indicated its persisting association with adult height.^13^ Large multinational cohorts have shown poor adult educational achievement in individuals stunted in early childhood.^14^ Childhood stunting is concurrently associated with elevated morbidity and mortality^2^ and poor child development^15^; and predictively associated with adult cognition, years of schooling and monthly income.^16^

Growth patterns in Indian children have been evaluated as a component of multisite multinational studies.^17^^–^^19^ A New Delhi-based cohort showed mean height-for-age z-score (HAZ) declining from −0.6 at birth to −1.9 at 24 months and subsequently unchanged in childhood.^17^^,^^20^ The Young Lives Indian cohort recruited from Andhra Pradesh had a mean length for age z-scores (LAZ) of −1.4 at 1 year of age.^19^ Recent study from Telangana found that infants aged 6–14 months and preschoolers aged 29–49 months of age had mean LAZ/HAZ of −1.1 and −1.8 respectively.^15^ A national level analysis of burden of malnutrition in Indian states reported that 39.3% of children younger than 5 years is stunted.^21^

Early childhood growth in urban Vellore low-income (slum) settings was evaluated in different birth cohorts—rotavirus cohort recruited between 2002 and 2003,^22^ and “The Etiology, Risk Factors and Interactions of Enteric Infections and Malnutrition and the Consequences for Child Health and Development (MAL-ED) Network” cohort recruited between 2010 and 2012.^18^ The current analysis evaluates the secular trend of early childhood growth patterns in low-income settings utilizing these two birth cohorts. Secular trends in growth could reflect the overall socio-cultural economic, and environmental changes in the community.^23^ Understanding these trends could help not only to chart community progress, but also to recognize the foundation on which further nutritional and health interventions could be planned. It is hypothesized that percentage of stunting will decline over time and socio-economic status (SES) will be the significant contributor for individual cohort growth trends and the change over time.

## MATERIALS AND METHODS

### Settings and subjects.

The current analysis used data from two birth cohorts recruited in urban low-income settings between 2002 and 2012 in Vellore. Vellore district situated in north-eastern Tamil Nadu in south India, has a total population of 3,936,331. It has a sex ratio of 1,004 females for 1,000 males in comparison to the national sex ratio of 943 females for 1,000 males and a literacy rate of 79.2% above the national average of 72.9%.^24^

### Rotavirus cohort.

The rotavirus birth cohort was recruited between March 2002 and August 2003 from three adjacent urban low-income settings in Vellore, namely Chinnallapuram, Ramnaickenpalayam, and Kaspa for studies on rotaviral diarrhea.^22^ All term newborns born in the recruitment period were included in the study with prior written informed consent from parents. Exclusion criteria included those living in brick-built houses with five or more rooms and children with very low birth weight (< 1,500 g). The recruited children had twice weekly home visits for collecting morbidity information, monthly anthropometric measurements and daily home visits in case of a diarrheal episode, till they completed 3 years of age. This birth cohort initially recruited 452 children.

### The MAL-ED cohort.

The MAL-ED birth cohort in India was recruited as one of the sites for a multinational, longitudinal prospective cohort study conducted in eight different countries across the world.^18^ The study site in India was in Old Town, and Salavanpet in Vellore and recruitment was between March 2010 and February 2012.^25^ All term newborn children were recruited immediately after birth with written informed parental consent. Exclusion included multiple pregnancy, pre-existing comorbidity in the child, very low birth weight, and another child of the same family being already enrolled in the MAL-ED study. Trained fieldworkers visited their home twice per week for active disease surveillance of the child and monthly anthropometric measurements were done for the first 2 years of life, with subsequent monthly follow-ups till 3 years of age. The initial birth cohort recruited 251 children. Details of recruitment, exclusion criteria, and follow-up are already published.^26^^–^^28^

Birth cohort studies and their subsequent follow-up were approved by the Institutional review board of Christian Medical College, Vellore.

### Anthropometric measurement.

Birth weight was taken from delivery records of children. Length/height of children was measured using an infantometer till 12 months of age or until the child was able to stand and subsequently with a wall-mounted tape in the rotavirus cohort.^22^ Length was measured using infantometer/measuring board till 24 months in the MAL-ED cohort and subsequently with a stadiometer.^18^^,^^29^ Weight was measured using a digital weighing scale in both cohorts to the nearest 10 g. The quality assurance of the anthropometric assessments was done by initial training of study personnel, regular calibration of instruments, quarterly retraining of study personnel, and standardization of the procedures.

### Socio-economic status.

The SES was assessed using a five-point scale, a modified version of the Kuppuswamy’s urban SES scale in the rotavirus cohort.^22^^,^^29^ The components of the scale included the number of rooms in the house, house ownership, at least one household member having finished high school education, occupation of the head of the household, and the number of material possessions. The final score of this scale ranged from 1 to 5.

In the MAL-ED study, SES was assessed using the WAMI measure, which included access to improved **W**ater and sanitation, **A**ssets, **M**aternal education, and total household **I**ncome.^30^ Information about the type of water source and latrine facility was included in “Water and sanitation.” Assets were calculated using the total number of physical assets such as telephone, television, vehicle, etc. The WAMI questionnaire was translated to Tamil, back-translated, and piloted prior to its administration by a trained field worker. The final measure calculated from these variables was converted to a standardized score ranging from 0 to 1. We used the median to classify the scores into high and low SES in both the studies.

### DIARRHEA.

An episode of diarrhea was defined as three or more watery stools in a 24-hour period or, in breast-fed children, more daily stools considered to be diarrhea by the mother.^31^^,^^32^ For the current analysis, the number of episodes of diarrhea during the first 3 years was estimated from both cohorts.

### **Education of head of the household and maternal education**.

The highest completed education status of the head of the household and mother was collected in different categories namely no education, primary schooling, secondary schooling, high schooling, higher secondary schooling, degree, and above. This information was later categorized into high education defined as secondary schooling and above and low education comprising of no education and primary schooling.

### Data entry and analysis.

Both studies had double entry database in place and the data was routinely monitored by data management team throughout the follow-up before locking the database. Additionally, in the MAL-ED cohort, the database was managed by the central Data Co-ordinating center of the MAL-ED study.^18^

### Statistical analysis.

Monthly anthropometric data was extracted from respective databases. Length measured for children ≤ 24 months were converted to LAZ and height measured for children > 24 months converted to HAZ scores using the WHO anthro-software growth standards.^33^ The distribution of the baseline socio-demographic variables was presented using count (percentage), mean (Standard Deviation), and median (Inter Quartile Range). Households with less than median socio-economic score for each cohort were categorized as low SES.

Proportions were compared using χ^2^ test, whereas Wilcoxon rank-sum test was used for comparing the continuous variables. We modeled LAZ/HAZ score over time (time in months). Since the LAZ/HAZ for each child measurements were clustered/correlated at the level of individual child we introduced a random intercept indicating individual child. This allows the model to have different intercept for each child, thus accounting for the clustering/correlation of LAZ/HAZ score for each child. Subsequently low birth weight, gender, SES, education of the head of the household and firstborn child in the family were included as fixed effect in the model. The equation of the model is as follows:

$${\text{HAZ}}_{ij}={\text{ß}}_{0}+{\text{ß}}_{1}{\text{LBW}}_{ij}+{\text{ß}}_{2}{\text{SES}}_{ij}+{\text{ß}}_{3}{\text{SEX}}_{ij}+{\text{ß}}_{4}{\text{EDU}}_{ij}+{\text{ß}}_{5}{\text{FIRSTCHILD}}_{ij}+{\text{ß}}_{6}{\text{MONTH}}_{ij}+{\text{V}}_{ij}+{\text{E}}_{ij}$$

${\text{Y}}_{ij}<$ is the response for the *j*-th measurement of the *I*-th subject

${\text{ß}}_{0}<$ is the fixed slope for the regression model

${\text{ß}}_{0}{\text{,}\;\text{ß}}_{1}{\text{,}\;\text{ß}}_{3}{\text{,}\;\text{ß}}_{4}{\text{,}\;\text{ß}}_{5}{\text{,}\;\text{ß}}_{6}$ are the fixed slope for the regression model for low birth weight, SES, female gender, education of head of household, first child in household, and months respectively. Month is the age of the child in months at the time of each measurement and ranged from 0 to 36 months.

${\text{X}}_{ij}<$ is the predictor of the *j*th measurement of the *i*th subject

${\text{V}}_{ij}<$ is the random intercept for the *i*th subject

${\text{E}}_{ij}<$ is the error term

Akaikie information criterion (AIC) was used for finalizing the model. We started with a combined model with variables: low birth weight, gender, SES, education of the head of the household, first born child, and total number of diarrhea in 3 years. The variable with least effect size was removed for the model in a sequential order and the AIC value for each model was compared until the AIC value started to increase. The model with the lowest AIC value was presented. We presented data for the effect of factors for individual studies separately. STATA version14.2 StataCorp. 2015. *Stata Statistical Software: Release 14* (StataCorp LP, College Station, TX), was used for performing the statistical analysis. We used “mixedpower” R package for performing the power analysis.

## RESULTS

**Figure 1.**
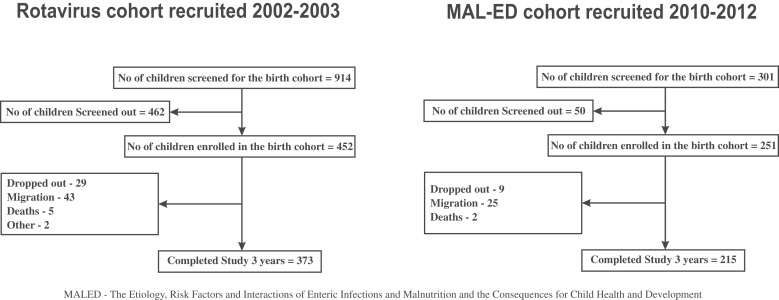
Follow-up of birth cohorts.

In the rotavirus cohort, 373 children completed the 3-year follow-up from the original cohort of 452 (82.5%). The MAL-ED cohort at 3 years had 215 children out of the original birth cohort of 251 (85.7%). The major reason for loss to follow-up was migration of families out of the study area (Figure 1). Baseline characteristics of cohorts are provided in Table 1 and both cohorts were comparable in birth weight and sex distribution. The MAL-ED cohort had better SES markers and mothers had more years of formal education. The MAL-ED cohort had higher number of episodes of diarrhea when compared with the rotavirus cohort.

**Table 1:** Baseline characteristics of children from both birth cohorts who completed 3 years of follow up (*N* = 588)

	Rotavirus study 2002 (*N* = 373)	MAL-ED study 2010 (*N* = 215)	*P* value
	n (%)	n (%)	
Birth weight*	2.91 (0.43)	2.89 (0.45)	0.646
Low birth weight	43 (11.8)	36 (17.1)	0.072
*Sex*			
Male	186 (49.9)	99 (46.0)	0.372
Female	187 (50.1)	116 (54.0)	
First born child in family	118 (31.6)	70 (32.6)	0.817
*SES*			
High SES	144 (38.6)	106 (49.3)	0.012
Low SES	229 (61.4)	109 (50.7)	
*Education of head of household*			
High education	170 (45.6)	85 (39.5)	0.155
Primary school and below	203 (54.4)	130 (60.5)	
*Education of the mother*			
High education	158 (42.4)	134 (62.3)	< 0.001
Primary school and below	215 (57.6)	81 (37.7)	
*Diarrhea during first three years*			
Number of episodes†	4 (2–7)	7 (4−10)	< 0.001
< 5 episodes	195 (52.3)	61 (28.4)	< 0.001
five or more episode	178 (47.7)	154 (71.6)	
*Diarrhea during first year of life*			
Number of episodes†	2 (1–4)	3 (2-6)	< 0.001
< 3 episodes	199 (53.4)	75 (34.9)	< 0.001
Three or more episode	174 (46.6)	140 (65.1)	

SES = socio-economic status.

* Mean (SD).

† Median (IQR).

MAL-ED—The Etiology, Risk Factors and Interactions of Enteric Infections and Malnutrition and the Consequences for Child Health and Development Network

The mean height (SD) of children at 3 years of age in the rotavirus and MAL-ED cohorts was 87.5 and 93.6 cm respectively, with corresponding HAZ scores of −2.20 and −1.92 significantly different (*P* = 0.0006). Supplemental Figure 1a compares HAZ scores between both cohorts. At birth recruitment, both cohorts had comparable LAZ scores: mean LAZ score in rotavirus and MAL-ED cohorts being −0.99 and −0.98 respectively (*P* = 0.9415). At 1 and 2 years of age, children in the rotavirus cohort had significantly lower LAZ than those in the MAL-ED cohort. Comparison of proportion of stunted children in these cohorts also showed similar trends (Supplemental Figure 1b). In the rotavirus cohort, 46.6, 67.1, and 63.2% of children were stunted at 1, 2, and 3 years of age respectively. Meanwhile in the MAL-ED cohort, significantly lesser proportion of children was stunted at 1, 2, and 3 years of age (31.5, 44.9, and 45.3% respectively).

Univariate analysis between diarrhea and LAZ/HAZ scores did not show any significant association and thus not included in the final model. The linear mixed effects model evaluating the linear growth during the first 3 years of age showed that low birth weight and being a female child were risks for stunting in both cohorts (Table 2). There was no association between SES and stunting in the rotavirus cohort, while SES was associated with linear growth in the MAL-ED cohort. The association of education of the head of household with linear growth as seen in the rotavirus cohort was absent in the MAL-ED cohort. Power analysis was done using “mixedpower” R package (Supplemental Figure 2). As outlier analysis showed no change in estimate (Supplemental Table 1), the effect of outliers was excluded.

**Table 2:** Estimates from the linear mixed effects model for the effect of factors associated with linear growth during the first 3 years of life (height-for-age “z” scores) for each cohort

	Rotavirus study 2002				MAL-ED study 2010			
	Unadjusted β	*P* value	Adjusted β	*P* value	Unadjusted β	*P* value	Adjusted β	*P* value
Low birth weight	−0.80 (−1.08, −0.52)	< 0.001	−0.76 (−1.04, −0.49)	< 0.001	−0.37 (−0.67, −0.08)	0.013	−0.43 (−0.72, −0.15)	0.003
Female	0.30 (0.12, 0.48)	0.001	0.28 (0.11, 0.46)	0.001	0.35 (0.13, 0.56)	0.002	0.35 (0.14, 0.56)	0.001
Low SES	−0.14 (−0.33, 0.05)	0.147	−0.06 (−0.24,0.12)	0.541	−0.30 (−0.52, −0.08)	0.007	−0.27 (−0.48, −0.05)	0.015
Low education of head of household	−0.36 (−0.55, −0.18)	< 0.001	−0.25 (−0.43, −0.07)	0.006	−0.18 (0.40, 0.05)	0.126	−0.15 (−0.37, 0.07)	0.175
First born child in the family	0.07 (−0.13, 0.26)	0.510	0.16 (−0.03, 0.35)	0.094	0.17 (−0.06, 0.41)	0.149	0.16 (−0.07, 0.39)	0.163
Month	−0.0344 (−0.036, −0.03394)	< 0.001	−0.04 (−0.04,−0.04)	< 0.001	−0.03 (−0.03, −0.02)	< 0.001	−0.03 (−0.03, −0.03)	< 0.001

SES = socio-economic status.

MAL-ED—The Etiology, Risk Factors and Interactions of Enteric Infections and Malnutrition and the Consequences for Child Health and Development Network

## DISCUSSION

The current analysis evaluated early childhood growth trends in two low-income birth cohorts recruited 8–9 years apart in Vellore, India. The mean HAZ at 3 years of age improved over this time period with corresponding reduction in proportion of stunting. Risks for stunting in both cohorts were low birth weight and being a female child. Socio-economic status did not have an independent association with growth in the rotavirus cohort, while had an association in the MAL-ED cohort even after correction with other factors. Both SES and maternal education were better in the MAL-ED cohort, when compared with the previous rotavirus cohort.

As per the 2020 Global Nutrition Report, stunting and wasting are highly prevalent in south Asia especially India,^34^ echoing previous international findings.^3^ As early childhood stunting is associated with concurrent mortality and morbidity, as well as adult-onset diseases, cognitive ability, and economic potential,^2^ there have been concerted efforts to understand trends and predictors of stunting to promulgate a framework for future intervention.^3^^,^^21^^,^^35^^,^^36^ The current analysis adds to the existing literature by evaluating trends in an urban low-income setting in south India over a decade utilizing two birth cohorts with good early childhood data granularity. Despite improvements in SES, the persistence of high prevalence of birth and early childhood stunting highlights a public health exigency to optimize maternal and early childhood nutrition.

Both birth cohorts retained more than 80% children at 3-year follow-up and the main reason for loss to follow-up was migration out of the study area (Figure 1), as families moved out to explore better vocational, educational, and living experiences. Parameters such as birth weight and sex distribution of both cohorts were comparable. Socio-economic status and maternal education improved in the MAL-ED cohort, despite educational level of the head of the household, usually a male member, remaining comparable to that of the rotavirus cohort. In the MAL-ED cohort itself, our previous publication has shown that SES improved between 6 and 36 months of age due to gains in water and sanitation access, assets, and reported family income.^26^ These SES improvements are in concurrence with economic surveys and reports from India and the World Bank indicating similar improvements in SES all over the world specifically in southern India where Vellore is situated.^36^^–^^38^

Children in both cohorts had mean LAZ around −1 at birth, worse than −0.6 reported at birth in New Delhi by the Consortium on Health-Orientated Research in Transitional Societies (COHORTS) collaboration.^17^ Affluent Indian children are shown to have growth potential similar to global standards.^39^ Despite improvements in SES and maternal education, the low birth length may be due to the high prevalence of maternal malnutrition—20% of antenatal mothers was underweight (body mass index < 18.5) and 21% overweight (body mass index ≥ 25) in the MAL-ED cohort.^25^^,^^40^ The mean LAZ/HAZ falling till 2–3 years of age as shown in our cohorts has been reported from other Indian studies.^15^^,^^19^ Improvements in proportion of stunting in early childhood between these two cohorts with similar low-income settings, concur with similar findings of improved stunting rates in India^21^^,^^36^ and the world.^1^^,^^3^

Though MAL-ED children had more early childhood diarrheal illnesses than rotavirus children, diarrhea did not contribute to the risk of stunting in both cohorts. Diarrheal episodes are shown to affect growth in individual studies and pooled analysis.^9^^–^^11^ An analysis of predictors of stunting in the large MAL-ED cohort of eight countries showed exposure to diarrheal pathogen a risk factor, not the number of diarrheal episodes,^7^^,^^12^ and low nutritional intake especially of energy and protein-rich food another risk factor.^12^ It is possible that MAL-ED cohort had better nutritional access, as their families had better SES and their mothers had better education, thus negating the higher incidence of diarrheal episodes.

In the current analysis, SES was associated with early childhood linear growth in the MAL-ED cohort, but not in the rotavirus cohort. Measurements of SES have evolved over time and the WAMI measure used in the MAL-ED study was selected after comparing three other approaches^30^ and might be more robust than the Kuppuswamy’s urban SES scale used in the rotavirus study.^22^ Improved SES is associated with not just improved childhood linear growth,^4^^,^^41^ but also development^42^ and cognition.^43^^,^^44^ A previous analysis of trends of childhood development milestones in the MAL-ED India cohort showed that SES was associated with cognition and language scores.^45^ Better SES as seen in the MAL-ED cohort can improve stunting through better sanitation, improved nutrition, and less debilitating infections.

Birth weight was a predictor of early childhood linear growth in both cohorts and is in concurrence with pooled data analysis showing that fetal growth restriction was the biggest contributor to stunting across 137 developing countries.^4^ Being a female child was associated with a lower linear growth in early childhood in both cohorts, probably due to female child discrimination common in India.^46^ However, there has been a recent trend reported in analyses that males are more vulnerable to health inequalities and stunting in early childhood probably due to better nutrition in females.^47^^,^^48^

Stunting has been recognized as an urgent priority in India.^3^^,^^36^ The National Nutrition Mission (NNM) launched by the Indian Government aims to eradicate malnutrition in India by 2022.^49^ Nutritional support for pregnant mothers and young children can improve the HAZ decline as seen in both our cohorts. Extending the Balwadi program that currently caters to preschool children aged 3 years onwards^50^^,^^51^ to infants and toddlers will also help this cause. Meanwhile, overall improvements in maternal education status and SES status should be also targeted. Other nonnutritional sector interventions including maternal education are shown to contribute around 50% to improvements in childhood stunting in evaluations across countries.^35^

Both studies were looking at diarrheal diseases and had similar protocols in terms of study inclusion, exclusion criteria, and assessment of anthropometric measurements. There was difference in definitions of certain variables such as the SES, however, these are unlikely to have affected the prevalence of stunting in either cohort. Both cohorts had similar mean sex ratio (*P* value 0.372) and birth weight (*P* value 0.646) suggesting cohorts were comparable. The prevalence of stunting during early childhood was lower among the MAL-ED cohort when compared with the rotavirus study cohort. This happened despite more children in the MAL-ED cohort experiencing a higher cumulative burden of diarrhea.

Cohorts were set up in a semi urban slum setting. Hence houses coming under the definition of mansions (brick-built houses with five or more rooms) were excluded from the rotavirus study. Similar house was not present in the second study area. The house with largest number of rooms in the MAL-ED cohort had four rooms. On an average the number of rooms in the house was higher in rotavirus cohort when compared with the MAL-ED house (1.67 versus 1.39 *P* value < 0.001).

If the difference in the growth trend was just because of the difference in the study population, then MAL-ED cohort should have experienced higher burden of stunting when compared with the rotavirus cohort. However, we observed that despite of being disadvantaged with higher burden of infection the MAL-ED cohort performed better than the rotavirus cohort because of the underlying secular trend in the improvement of child nutrition across the state during the time period.

The original cohort collected information on all morbidity from children and classified it into diarrheal disease, respiratory illness, other infectious disease, noninfectious disease, and fever. However, the focus of both studies was on diarrheal episodes, which were followed up with appropriate investigations. Both studies had hypothesized that enteric infections might affect the linear growth of children during early childhood.

Limitations of the current study include comparatively smaller sample sizes of our cohorts and the loss to follow up. Nutritional details were available only for the MAL-ED cohort, limiting an exploratory comparison. As per our knowledge, this is the first study from India comparing two birth cohorts recruited almost a decade apart in low-income settings in one urban area. Both MAL-ED and rotavirus cohorts had standardized assessments with rigorous quality control measures and good early childhood data granularity. In addition, the data collected by field workers were validated in a 10% random subsample on revisits by the study supervisor and/or the physician.

Despite limitations, the current analysis showed improvements in HAZ and proportion of early childhood stunting between two birth cohorts recruited 8–9 years apart in low-income settings in urban Vellore in south India, the later-recruited taller cohort having better SES and maternal education. Stunting was associated with SES in the later cohort even after corrections. Future studies could incorporate nutritional and nonnutritional interventions in vulnerable populations to evaluate their effect on birth weight as well as early childhood stunting.

## Supplemental Material


Supplemental materials
