# Supplementary material for: Secular Growth Trends in Early Childhood—Evidence from Two Low-Income Birth Cohorts Recruited over a Decade in Vellore, India
Source: Am J Trop Med Hyg. 2022 Jul 13;107(1):45–51. doi: 10.4269/ajtmh.21-0886 (PMC9294682; doi:10.4269/ajtmh.21-0886)
Supplement: Supplementary file 1 [file tpmd210886.SD1.pdf]

Supplemental Figure 1a

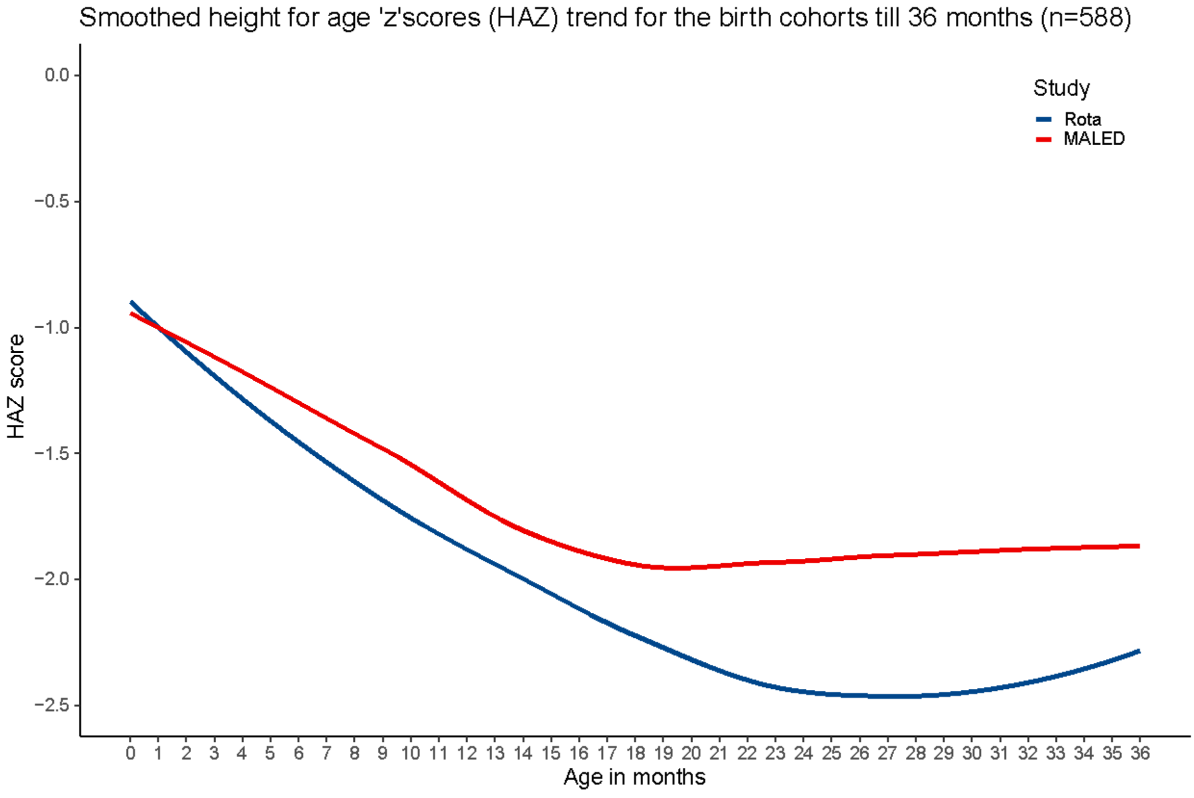

Supplemental Figure 1b

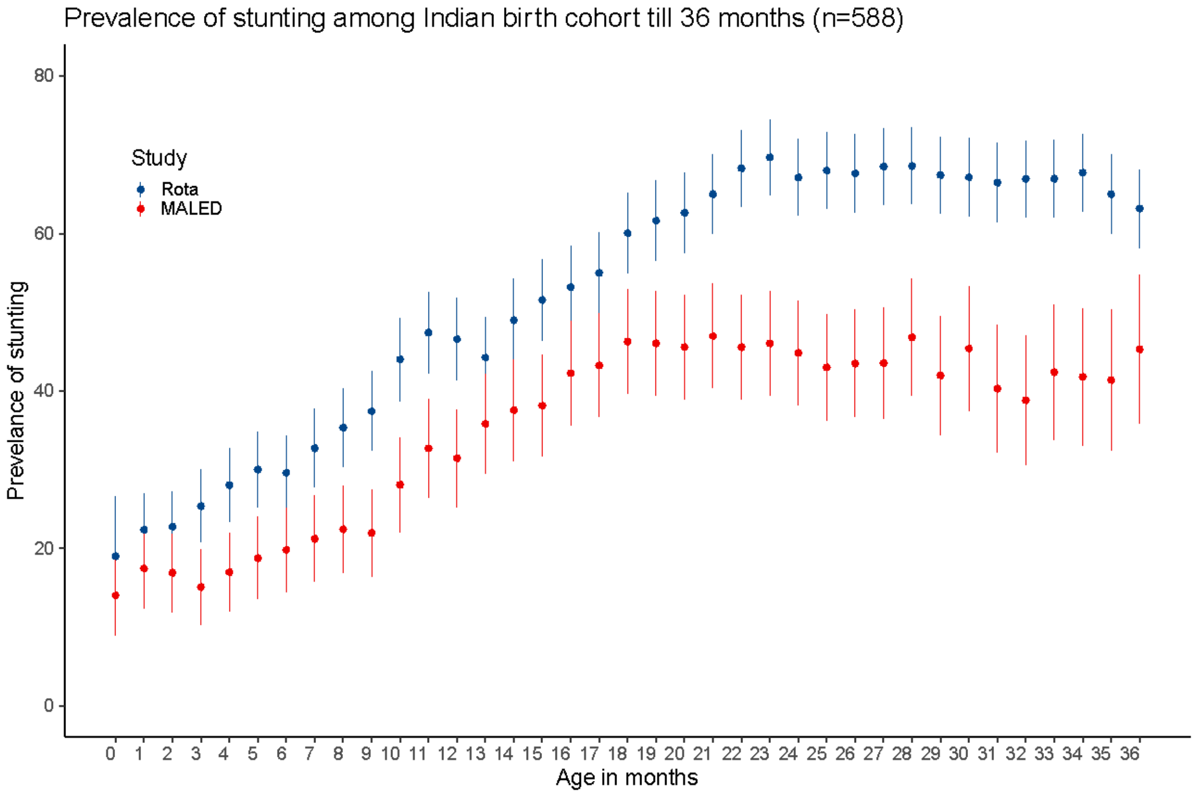

Supplemental Figure 2. Power analysis for the linear mixed model for Height for age z score for both cohorts.

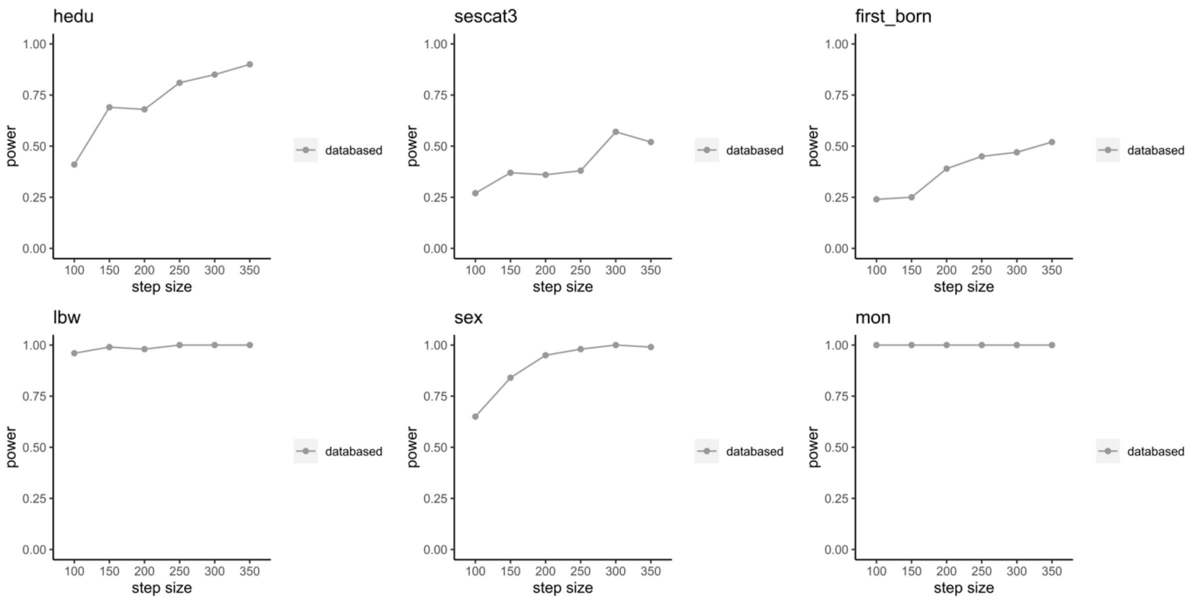

Supplemental Table 1. Outlier analysis

Height for age 'z'score	Rotavirus study		MALED study	
	**Adjusted ß**	***P*-value**	**Adjusted ß**	***P*-value**
Low birth weight	-0.75 (-1.02, -0.49)	<0.001	-0.46 (-0.74, -0.17)	0.002
Female gender	0.26 (0.10, 0.43)	0.002	0.33 (0.12, 0.54)	0.002
Low SES	-0.07 (-0.24, 0.10)	0.436	-0.32 (-0.54, -0.10)	0.005
Low education of head of household	-0.25 (-0.42, -0.08)	0.004	-0.16 (-0.38, 0.05)	0.143
First born child in the household	0.16 (-0.02, 0.34)	0.078	0.17 (-0.06, 0.39)	0.152
Age in months	-0.04 (-0.04, -0.04)	<0.001	-0.03 (-0.03, -0.03)	<0.001
